# Indications and the requirements for single-use medical gloves

**DOI:** 10.3205/dgkh000261

**Published:** 2016-01-12

**Authors:** Axel Kramer, Ojan Assadian

**Affiliations:** 1 Institute of Hygiene and Environmental Medicine, University Medicine Greifswald, Germany; 2 Institute for Skin Integrity and Infection Prevention, University of Huddersfield, United Kingdom

**Keywords:** single-use medical gloves, indications, requirements, definitions, “germ-poor” single-use gloves, pathogen-free single-use gloves

## Abstract

**Aim:** While the requirements for single-use gloves for staff protection are clearly defined, the conventional medical differentiation between “sterile surgical gloves” used during surgical procedures and “single-use medical gloves” used in non-sterile medical areas does not adequately define the different requirements in these two areas of use. Sterilization of single-use medical gloves is not performed if sterility is not required; thus, another terminology must be found to identify the safety quality of non-sterile single-use medical gloves. Therefore, the labeling of such gloves should reflect this situation, by introducing the term “pathogen-free” single-use glove. The hygienic safety of such a glove would be attainable by ensuring aseptic manufacturing conditions during manufacturing and control of pathogen load of batch controls after fabrication.

**Proposed recommendation:** Because single-use gloves employed in non-sterile areas come into contact not only with intact skin but also with mucous membranes, no potential pathogens should be detectable in 100 mL of rinse sample. In order to declare such gloves as pathogen-free we suggest absence of the indicator species *S. aureus* and *E. coli*. In addition, the total CFU count should be evaluated, since a high load indicates lack of optimal hygiene during the manufacturing process. Based on the requirements for potable water and findings obtained from investigations of the bacterial load of such gloves after manufacturing, the here suggested limit for the total bacterial count of <10^2^ CFU/mL of rinse sample per glove seems realistic.

## Zusammenfassung

**Problemstellung:** Während die Anforderungen an Einmalhandschuhe für den Personalschutz definiert sind, trägt die in der Medizin gängige Unterscheidung zwischen „sterilen chirurgischen Operations- (OP)- Handschuhen“ und „medizinischen Einmalhandschuhen“ zur Anwendung in nicht sterilen Bereichen den unterschiedlichen Anforderungen bezüglich der Erregerbelastung nur ungenügend Rechnung. Da aus Kostengründen, aber auch aufgrund fehlender hygienischer Notwendigkeit keine abschließende Sterilisation medizinischer Einmalhandschuhe durchgeführt wird, sofern keine Sterilität benötigt wird, muss eine andere Lösung gefunden werden, um die Unbedenklich nicht abschließend sterilisierter medizinischer Einmalhandschuhe zu gewährleisten und auszuweisen. Zugleich sollte die Bezeichnung der Handschuhe diesem Umstand Rechnung tragen. Letzteres wäre durch die Einführung des Terminus „pathogenfreier“ Einmalhandschuh gegeben. Die hygienische Unbedenklichkeit eines derartigen Handschuhs wäre durch die Gewährleistung aseptischer Herstellungsbedingungen erreichbar und durch eine Chargenkontrolle nach der Herstellung in Bezug auf die Erregerbelastung nachweisbar.

**Anforderungen:** Da Einmalhandschuhe zur Anwendung in nicht sterilen Bereichen nicht nur mit intakter Haut, sondern auch mit Schleimhäuten in Kontakt kommen, sollten in 100 mL Sammelflüssigkeit keine potentiell pathogenen Erreger nachweisbar sein. Für die Deklarierung der Eigenschaft pathogenfrei („keimarm“) wird die Beschränkung auf die Indikatorspecies *S. aureus* und auf *E. coli* vorgeschlagen. Obwohl die Gesamtkoloniezahl für den Patientenschutz nicht relevant ist, erscheint auch ihre Bestimmung sinnvoll, weil eine hohe Belastung auf hygienische Mängel im Herstellungsprozess hinweist. Unter Berücksichtigung der Anforderungen an Trinkwasser sowie der erzielten Befunde erscheint ein Grenzwert von <10^2^ KbE/mL Sammelflüssigkeit bezogen auf einen Handschuh realistisch.

## Introduction

In health-care facilities, medical staff uses gloves for the following indications:

- Protection of the wearer from contamination with blood, secretions, and excretions and the associated risk of contamination with pathogens capable of reproduction
- Prevention of pathogen release from the hand into the sterile work area during aseptic duties
- Protection from chemicals
- Defined pathogen barrier as protection from biological agents
- Radiation protection
- Textile undergloves employed to reduction of amount of sweat produced in the glove.

These various indications demand different qualities of the gloves. 

For the first four indications, single-use gloves made of latex or synthetic material are employed single or double. In contrast, to reduce the amount of sweat produced by prolonged wearing of such gloves, textile (e.g. cotton) undergloves are worn which, after wearing, can be processed for further use by performing a disinfection wash cycle [1]. However, sterile undergloves must be worn under sterile surgical gloves. 

While the requirements for single-use gloves for staff protection are clearly defined, the conventional differentiation between “sterile surgical gloves” used for surgical procedures and “single-use medical gloves” used in non-sterile areas does not adequately define the different requirements in the two areas of use. In particular, the currently used German description of non-sterile single-use gloves as “germ-poor” disposable gloves is merely a broad qualitative description, and not quantitatively objectified. The term “germ poor” used in German speaking countries only means that no risk of infection emanates from such “germ-poor” objects. However, it is not clear whether this property – should it exist at all – is ensured by low levels of pathogens or the possible presence of largely non-virulent microorganisms, and which requirements result from this, since the infection risk depends on multiple factors, such as glove use, pathogen load, and the virulence of microorganisms present. Moreover, the term “germ-poor” is used only in German; it does not occur in other languages. Nevertheless, the English term “clean single-use gloves” or the French term “gants d’examen” (examination gloves) also do not solve the problem. Even the expression “not sterile”, used by Hughes et al. [2], says nothing about the contamination risk of such gloves. Because – in analogy to the other descriptions mentioned here – the term “germ-poor single-use glove” says nothing about the actual microbial load or possible risks of infection upon employment in the various areas of use, this article suggests and explains a new proposed terminology “pathogen-free single-use gloves” which shall better reflect the required properties of such gloves.

## Distinction between gloves as a medical device (MDD) and gloves as personal protective equipment (PPE), and resulting requirements

### Requirements for single-use gloves declared as MDD

In terms of protection against infection, sterile surgical gloves should meet infection protection requirements in both directions, i.e., protection of the patient and the wearer. 

In contrast, single-use gloves that are not intended for sterile use serve only to protect the wearer from contamination. It is a common misconception that these non-sterile single-use gloves, used correctly, also serve to protect the patient. However, they do provide some indirect protection by preventing massive contamination, so that conventional hand disinfection after removing the gloves is sufficient to guarantee that any residual pathogens on the skin will be killed. For instance, after artificial massive contamination with *E. coli* approximately 2 log_10_ remained on the hands after hand disinfection [3]. Analogous data exist for MRSA, where 2–3 log_10_ residual contamination of the hands remained after hand disinfection [4]. Thus, single-use gloves can also facilitate the interruption of infection transmission [5], [6], [7], if used adequately. Based on their intended function, both types of gloves are classified as medical devices. 

In addition to the physical requirements, surgical gloves must also be sterile [8]. Furthermore, the endotoxin content must not exceed 20 endotoxin units per pair of gloves if gloves are labeled as having “low endotoxin content” [9], [10]. The content of powder must not exceed 2.0 mg per glove to qualify them as powder-free gloves [11]. The manufacturer shall monitor the leachable protein in finished gloves containing natural rubber latex by the method specified in EN 455-3 [10].

Single-use gloves intended for use in non-sterile areas must meet the following requirements in order to fulfill their physical protection function [12]:

- Labeled as “MDD 93/42/EEC”
- AQL (accepted quality assurance level) of ≤1.5 in accordance with EN 455-1 [13]
- Tearing strength during production of at least 9 Newton in accordance with EN 455-2 [14]
- Biocompatibility in terms of chemicals, endotoxins, and freedom from powder and leachable proteins in accordance with EN 455-3 [10]
- Shelf life of at least 3 years in accordance with EN 455-4 [15].

However, this list does not mention requirements for the microbiological safety of single-use gloves employed in non-sterile applications.

Both in the Recommendations of the Commission for Hospital Hygiene and Infection Prevention of the Robert Koch Institute (KRINKO) Berlin and in the Guidelines of the Association of the Scientific Medical Societies in Germany (AWMF), the term “germ-poor single-use medical gloves” is used for single-use medical gloves employed for non-sterile applications. This characterization, however, is inadequate, since the qualitative term “germ poor” has neither been defined nor tested. To date, although these products are certified based on the MDD or PPE regulations, the pathogen load limit of these single-use gloves has not been tested. 

Because it is hygienically unnecessary and would incur needless expenses, final sterilization of single-use medical gloves is not performed if sterility is not required; thus, another solution must be found to ensure the safety of single-use medical gloves that do not undergo final sterilization. At the same time, the labeling of these gloves should reflect this situation, for instance, by introducing the term “pathogen-free” single-use glove, i.e., a glove that does not have to be sterile but must not be contaminated with pathogens. The hygienic safety of such a glove would be attainable by ensuring aseptic manufacturing conditions, and provable in terms of pathogen load by batch control after fabrication.

To investigate the current microbiological quality of single-use gloves employed in non-sterile applications, a microbiological examination of a representative sample (Ansell^®^ single-use glove, glove age 3–6 months, from each of 6–8 different factories and 4 different countries, total n=30) was conducted by an independent external laboratory (BMA, Bochum, Germany). A total of 11 different glove models were tested, comprising 2 ethylene-vinyl-acetate models, one neoprene model, 3 latex models, and 5 nitrile glove models. In accordance with DIN EN ISO 11737-1 [16], the gloves were examined for bacteria and fungi as well as total microbial count in 15 mL of each rinse sample. The total microbial count showed an average of 5 CFU/15 mL of rinse sample (minimum = 0 KbE/15 mL, maximum = 36 KbE/15 mL), and thus was 100 times lower than the threshold value for potable water. Similar results were obtained in a second investigation of a different premium glove manufacturer (SafeDon^®^, 3 different latex models and one nitrile model). The testing following the same methodology as described in the DIN EN ISO 11737-1 [16] yielded a mean microbial total count of 8.4 CFU/15 mL (maximum: 13.5 CFU/15 mL) and for the nitrile model a mean total microbial count of 4 CFU/15 mL auf (maximum: 14.1 CFU/15 mL). Again, not only the mean values, but also the maximum CFU/mL counts ranged more than 100 times below the microbiological recommendations for potable water in Europe.

Upon aseptic removal of gloves (n=38) from their boxes at an orthopedic ward [2], the total load varied between 0 and 9.6×10^3^ CFU/glove. *Bacillus spp*. were present on 82% of samples, skin commensals on 50% with coagulase-negative staphylococci (CoNS) as the predominant species, and *E. faecalis*, *K. pneumoniae*, *Pseudomonas *spp. or *S. aureus* were recovered from 13% of samples. Significantly more skin commensals and pathogens were recovered from samples from days 3, 6, 9 than samples taken upon box opening [2]. 

Because single-use gloves employed in non-sterile areas come into contact not only with intact skin but also with mucous membranes – e.g., when performing oral hygiene in ventilated patients or during vaginal or rectal examinations – they should be free of potentially pathogenic microorganisms; the quantitative requirements can be based on drinking water limits as well as on requirements for non-sterile medical preparations used in the oral cavity, nose, and ear. For non-sterile medical preparations, the limit is 10^2^ CFU of total aerobic microorganisms/g or mL and 10^1^ CFU of total yeast and moulds/g or mL. *S. aureus* and *P. aeruginosa* should not be detectable in 1 g or 1 mL [17]. For *P. aeruginosa* in drinking water, the requirements are not as strict, i.e., not detectable in 100 mL [18], because as opposed to freshly sampled tap water, the risk of further increase exists in medical preparations. The limit for drinking water, 10^2^ CFU of aerobic bacteria/mL, is also higher. With reference to the requirements for non-sterile medical preparations used in the oral cavity, nose, or ear and to drinking water requirements, the safety of single-use gloves in terms of patient protection can be assumed if no potential pathogens are detectable in 100 mL of rinse sample. Despite the fact that *E. faecalis*, *K. pneumoniae*, *Pseudomonas *spp., and *S. aureus* were detected on gloves aseptically taken from their box for actual applications [2], we suggest restricting the declaration as pathogen-free (previous term in German: “germ-poor”) to one indicator species each as a typical representative of skin or intestinal flora, i.e., *S. aureus* and *E. coli*, respectively. If future studies should demonstrate that this selection is insufficient, the scope of the examination can be broadened at any time. If no potentially pathogenic microorganisms are among the total CFU, the latter is not relevant for patient protection. Nevertheless, it still makes sense to identify the microorganisms in the total CFU because a high load indicates lack of optimal hygiene during the manufacturing process. Taking the requirements for potable water and the findings obtained here into account, the suggested limit of <10^2^ CFU/mL of rinse sample per glove seems realistic. 

### Properties of single-use gloves declared as PPE

If the wearer is to be protected from chemical and physical risks as well as biological agents (e.g., during work in a microbiological laboratory or care of patients harboring highly pathogenic microorganisms), gloves declared as PPE must be used. To ensure sufficient personal protection of staff, single-use gloves must meet the following requirements [12]:

- Protective gloves as PPE against chemicals and microorganisms must meet not only the general requirements given in ISO 11193-1 [19], EN 420 [20] – especially in terms of innocuity, ergonomics, resistance to water penetration and pH values between 3.5–9.5 – and EN 455-3 [10] with a protein content <10 µg/g, but also the special requirements related to purpose, e.g., protection from chemicals and microorganisms in accordance with EN 374 [21], [22], [23] and EN 388 [24], as well as mechanical resistance.
- ASTM F1671-07 provides information on resistance to blood-borne pathogens, e.g., viruses [25].
- ASTM D6978-05 regulates testing the barrier function against cytostatic agents [26].
- If protection against chemicals is to be achieved, gloves for high-risk situations (category III of RL 686, recognizable by the CE labeling followed by a 4-digit number) are indicated [27]. For clinical applications, a minimum of PPE category II for protection against moderate risks must be selected. However, PPE category III provides greater protection as well as replicable quality (AQL), which is decisive for the protection expected.

It is recommended to demand a PPE-CE certificate from the manufacturer when acquiring PPE. Labeling as “PPE 89/656/EEC” identifies the product as PPE.

Up to 2010, single-use gloves in healthcare facilities could only be classified according to either the EU Directive on medical products 93/42/EEC or the EU Directive on PPE for Users 89/656/EEC, despite similar properties. Since 2010, the revised EU Directive 2007/47/EC allows dual labeling of products for dual purposes as MDD and PPE, as well as the respective dual CE labeling. Dual-purpose single-use gloves thus bear the CE label “PPE 89/686/EEC” and “MDD 93/42/EEC”. Single-use gloves that are neither MDD nor PPE, and thus do not meet the quality criteria of EN 455 and EN 374, should not be used in the vicinity of patients. 

## Further considerations for safe usage of hygienic single-use gloves

Even if hygienic single-use gloves fulfill the requirements described above, they do not guarantee patient safety if the following factors are not taken into consideration during and after use:

- A single-use glove may only be used during care of one and the same patient and must be removed after the given task has been completed. Changing gloves is usually correlated with the indications for hand disinfection, e.g., when switching from contaminated (suctioning secretions) to uncontaminated (operation of infusion system) tasks [28], [29].
- Disinfecting gloved hands should be the exception, for instance, in situations which demand frequent changing of gloves but experience shows that it is difficult to realize, or in which a change gloves interrupts the work flow, e.g., when switching back and forth between contaminated to uncontaminated tasks on the same patient. In the latter situation, the skin can be damaged if time is short and fresh gloves are put onto hands still moist with alcohol.
- After taking off the glove, hand disinfection must always be performed; wearing gloves does not guarantee complete protection of hands from contamination, because glove perforation may occur unnoticed and a risk of contamination may arise if the glove is improperly removed from the hand [29], [30].
- Due to the risk of damaging the skin and an increased risk of perforation [31], single-use medical gloves should only be put on clean, completely dry hands [32].
- Hand disinfection must be performed if gloves are not available in an automatic glove dispenser or a specially designed cardboard box which, upon removal of a glove, partially exposes the subsequent glove to an extent that enables it to be taken without touching the box or the other gloves.

The glove must meet the following conditions to be able to undergo disinfection [32]:

- The glove must be certifiably disinfectable (frequency, material tolerance, make of glove, disinfectant) or resistant to chemicals in accordance with EN 374 [22]. If the manufacturer does not provide such information, the user him/herself can test this using one of the intended gloves and the disinfectant by determining the wear perforation rate, for instance, after 5 rounds of hand disinfection as described in [13].
- The glove exhibits no visible perforations.
- The glove is not visibly contaminated with blood, secretions, or excretions.
- Because the perforation rate increases with duration of wear or after physically demanding tasks, gloves worn in the intensive-care area should be changed after a maximum of 15 min and every time a patient is washed, even if all other above-mentioned safety measures have been performed/criteria have been met [33].

## Notes

### Competing interests

The authors declare that they have no competing interests.
